# Vancomycin Powder Is Highly Cost-Effective in Total Ankle Arthroplasty

**DOI:** 10.1177/19386400221136374

**Published:** 2022-11-23

**Authors:** Hannah H. Nam, Brandon J. Martinazzi, Gregory J. Kirchner, Adeshina Adeyemo, Kirsten Mansfield, Kelly Dopke, Anna Ptasinski, Vincenzo Bonaddio, Michael C. Aynardi

**Affiliations:** Penn State College of Medicine, Hershey, Pennsylvania; Department of Orthopaedics and Rehabilitation, Penn State Health Milton S. Hershey Medical Center, Hershey, Pennsylvania; Penn State College of Medicine, Hershey, Pennsylvania; Department of Orthopaedics and Rehabilitation, Penn State Health Milton S. Hershey Medical Center, Hershey, Pennsylvania; Department of Orthopaedics and Rehabilitation, Penn State Health Milton S. Hershey Medical Center, Hershey, Pennsylvania; Penn State College of Medicine, Hershey, Pennsylvania; Penn State College of Medicine, Hershey, Pennsylvania; Penn State College of Medicine, Hershey, Pennsylvania; Department of Orthopaedics and Rehabilitation, Penn State Health Milton S. Hershey Medical Center, Hershey, Pennsylvania; Department of Orthopaedics and Rehabilitation, Penn State Health Milton S. Hershey Medical Center, Hershey, Pennsylvania

**Keywords:** total ankle arthroplasty, prosthetic joint infection, vancomycin powder

## Abstract

Prosthetic joint infection (PJI) is a costly and potentially fatal complication in total ankle arthroplasty (TAA). Some surgeons apply topical vancomycin powder to minimize the risk of infection during TAA procedures. The purpose of our study was to determine the cost-effectiveness of using vancomycin powder to prevent PJI following TAA and to propose an economic model that can be applied by foot and ankle surgeons in their decision to incorporate vancomycin powder in practice. Using our institution’s records of the cost of 1 g of topical vancomycin powder, we performed a break-even analysis and calculated the absolute risk reduction and number needed to treat for varying costs of vancomycin powder, PJI infection rates, and costs of TAA revision. Costing $3.06 per gram at our institution, vancomycin powder was determined to be cost-effective in TAA if the PJI rate of 3% decreased by an absolute risk reduction of 0.02% (Number Needed to Treat = 5304). Furthermore, our results indicate that vancomycin powder can be highly cost-effective across a wide range of costs, PJI infection rates, and varying costs of TAA revision. The use of vancomycin powder remained cost-effective even when (1) the price of vancomycin powder was as low as $2.50 to as high as $100.00, (2) infection rates ranged from .05 to 3%, and (3) the cost of the TAA revision procedure ranged from $1000 to $10 000.

**Levels of Evidence:** IV

## Introduction

Prosthetic joint infection (PJI) following total ankle arthroplasty (TAA) is a serious and costly complication. While thought to be uncommon, previous research has reported up to 4% of patients who underwent TAA had a diagnosis of, or required additional procedures for PJI.^1,2^ As such, patients who develop PJI may incur added hospital expenses.

The economic impact that PJI can have on the healthcare system is substantial. For example, annual hospital expenditures related to PJI after hip or knee arthroplasty are projected to reach $1.85 billion by 2030.^3^ This, in part, can be attributed to the increasing number of hip and knee replacements performed annually.^3^ While the economic impact of PJI after hip and knee arthroplasty is extensive, little research has explored the economic impact of PJI following TAA. However, given that the number of TAAs performed over the last 14 years has increased by almost 50%, it is reasonable to presume that there will be a subsequent rise in PJI-related associated costs.^4^

“The use of vancomycin powder remained cost-effective when the price was as low as $2.50 to as high as $100.00.”

To prevent PJI following TAA, some surgeons will use intraoperative adjuvants. One such adjuvant is topical vancomycin powder, which is known to reduce the risk of surgical site infection in spine surgery.^5^ However, the use of vancomycin powder in foot and ankle surgery is controversial.^6^ Furthermore, there is a paucity of literature exploring the cost-effectiveness of topically applied vancomycin powder in TAA.

Therefore, the purpose of this study was to perform a break-even analysis to determine the cost-effectiveness of topically applied vancomycin powder for preventing PJI after TAA. In doing so, we sought to present an economic model that can be used by any foot and ankle surgeon at their own institution. We hypothesized that topically applied vancomycin powder would be highly cost-effective in PJI prophylaxis.

## Methods

**Figure 1.**
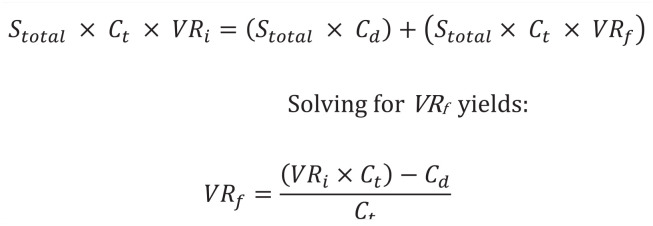
Equation used to calculate break-even PJI rate. Where: *S*_total_ = total annual surgeries; *C_t_* = total cost of TAA Revision; *C_d_* = cost of drug(s); *VR_i_* = initial PJI rate; *VR_f_* = break-even PJI rate. Adapted from Hatch et al.^7^ Abbreviations: PJI, prosthetic joint infection; TAA, total ankle arthroplasty.

The literature was searched to estimate the rate of PJI following TAA, as well as the estimated cost of TAA revision. The current literature does not have diagnostic criteria specific to PJI after TAA. For our literature search, we used the criteria outlined in the systematic review by Walley et al.^2^ We did not establish a time cut-off point following index procedure. Acute infection is defined as less than 4 weeks from index procedure while long-term infection is defined as greater than 4 weeks following the index procedure. We included both acute and long-term PJI for this study. Total ankle arthroplasty revision was defined as any procedure following the index procedure including irrigation and debridement, cement spacer placement, revision arthroplasty, and revision arthrodesis. Our institutional records were then queried to estimate the product cost of 1 g of topical vancomycin powder. A modified equation initially described by Hatch et al^7^ (Figure 1) was used to perform a break-even analysis. Our equation produces the final break-even rate necessary to make 1 g of topically applied vancomycin powder cost-effective. The difference between the initial and final break-even rate yields the absolute risk reduction (ARR). Using the ARR, the number of TAAs that could be performed while preventing a single PJI and still breaking even on cost was calculated (NNT). Additional sensitivity analyses were run to determine if vancomycin remained cost-effective at varying initial PJI rates and costs of TAA revision.

## Results

Costing $3.06 per gram at our institution, vancomycin powder was determined to be cost-effective in TAA if the PJI rate of 3% decreased by an ARR of 0.02% (NNT = 5304) (Table 1). Likewise, at this same initial PJI rate, varying the product cost for 1 g of vancomycin powder did not limit its ability to remain cost-effective (Table 1). The use of vancomycin powder remained cost-effective when the price was as low as $2.50 to as high as $100.00. Additional calculations at our institutional product cost were computed using varying initial PJI rates in an effort to reflect the variation among other surgeons and institutions. These calculations demonstrated that vancomycin powder remained cost-effective even if the initial PJI rate was as low as 0.05% (Table 2). Further analyses demonstrated that when holding the cost and initial rate of PJI constant, vancomycin remained cost-effective for TAA revision costs ranging from as low as $1000 to as high as $10 000 (Table 3).

**Table 1.** Vancomycin Powder Is Highly Cost-Effective in TAA.

Cost of vancomycin powder	Final break-even rate (%)	ARR (%)	NNT
$2.50	3.98	0.02	6492
$3.06^a^	3.98	0.02	5304
$10.00	3.94	0.06	1623
$17.00	3.90	0.10	955
$34.00	3.79	0.21	477
$44.00	3.73	0.27	369
$50.00	3.69	0.31	325
$75.00	3.54	0.46	216
$100.00	3.38	0.62	162

Assumes initial rate of PJI to be 4%. Assumes cost of revision TAA to be $16,230.

Abbreviations: TAA, total ankle arthroplasty; ARR, absolute risk reduction; NNT, number needed to treat.

a Cost of drug at our institution.

**Table 2.** Vancomycin Remains Cost-Effective Across a Wide Range of Initial Infection Rates.

Initial PJI rate (%)	Final break-even rate (%)	ARR (%)	NNT
0.05	0.03	0.02	5304
0.25	0.23	0.02	5304
0.5	0.48	0.02	5304
0.75	0.73	0.02	5304
1.0	0.98	0.02	5304
1.5	1.48	0.02	5304
2.0	1.98	0.02	5304
2.5	3.48	0.02	5304
3.0	2.98	0.02	5304

Assumes cost of revision TAA to be $16,230.^8^ Assumes cost of vancomycin powder to be $3.06.

Abbreviations: PJI, prosthetic joint infection; ARR, absolute risk reduction; NNT, number needed to treat.

**Table 3.** Vancomycin Powder Remains Cost-Effective at Varying Costs of TAA Revision.

Cost of TAA revision	Final break-even rate (%)	ARR (%)	NNT (%)
$1000.00	3.69	0.31	327
$2000.00	3.85	0.15	654
$3000.00	3.90	0.10	980
$4000.00	3.92	0.08	1307
$5000.00	3.94	0.06	1634
$6000.00	3.95	0.05	1961
$7000.00	3.96	0.04	2288
$8000.00	3.96	0.04	2614
$9000.00	3.97	0.03	2941
$10 000.00	3.97	0.03	3268

Assumes initial PJI rate of 4%. Assumes cost of drug to be $3.06.

Abbreviations: TAA, total ankle arthroplasty; ARR, absolute risk reduction; PJI, prosthetic joint infection; NNT, number needed to treat.

## Discussion

Intraoperative vancomycin powder has been shown to reduce the overall incidence of PJI in a number of orthopaedic subspecialities.^9-11^ According to a study published by Wukich et al,^12^ topically applied vancomycin powder significantly reduced the rate of surgical site infection in diabetic patients undergoing reconstructive foot and ankle surgery. The overall likelihood of deep surgical site infection was decreased by 73% in patients who received topically applied vancomycin (*P* = .0188) compared to control.^12^ Studies involving the use of topical vancomycin powder in foot and ankle surgery is severely limited, and further research regarding this topic is needed. This study is the first to evaluate the economic viability of topically applied intraoperative vancomycin during TAA. As the number of TAAs performed annually continues to increase,^12^ the incidence of PJI will likely follow. Therefore, mitigating infection risk while also reducing cost to the patient and health system is becoming increasingly important.

We found that given its low product cost, topically applied intraoperative vancomycin powder can be highly cost-effective at preventing PJI following TAA. Furthermore, at our institutional product cost, vancomycin remained cost-effective even at initial PJI rates as low as 0.05%. In addition, when the initial rate of PJI was held constant at 4%, vancomycin powder remained cost-effective across a wide range of prices. Finally, the cost of revision surgery did not impact the ability of vancomycin powder to remain cost-effective.

While our findings demonstrate vancomycin powder is highly cost-effective, our cost analysis may have potential flaws. First, we defined the cost of our adverse outcome to be revision surgery. However, in reality, a patient who develops a PJI after TAA will incur other added hospital and nonhospital-related expenses in addition to the cost of the revision. Thus, we likely underestimate the true economic burden that a PJI will have on the patient and health system. Second, while rare, we did not consider the cost of treating a complication that could arise as a consequence of vancomycin powder. For example, one study found that topical application of vancomycin powder impaired osteogenic differentiation of human mesenchymal stromal cells, reducing bone formation and affecting the healing process of fractures and arthrodesis.^13^ Furthermore, the potentially detrimental local effects of vancomycin powder in the skin healing process were not taken into consideration in this study. These complications were not calculated in our risk reduction model. Finally, our modeling only accounts for averages and does not capture patient-specific factors, such as demographics and comorbid conditions.

Despite these limitations, we believe that our analysis is useful as it provides data that would be otherwise unattainable in a larger clinical study. For example, assuming a theoretical ARR of 0.02%, 5304 patients would need to be treated to prevent a single PJI. To detect this same result in a large prospective clinical study, a power analysis demonstrates that the sample size would need to be 153 666 400, assuming a *P* < .05 and power = 80%. Furthermore, the utility of this break-even analysis is that it can be readily applied by surgeons at their own institutions. They simply need the initial institutional PJI rates, cost of vancomycin powder, and estimates of the cost of revision surgery or PJI treatment. In doing so, they will be able to determine if the use of vancomycin powder is cost-effective within their own practice.

While more evidence is needed to determine the true efficacy of vancomycin powder during TAA, the cost-effectiveness of the drug should not be ignored. Furthermore, surgeons should also be mindful of the economic burden adverse outcomes like PJI can have on both the healthcare system and the patient. This study demonstrates that intraoperative vancomycin powder can be highly cost-effective and should be favorably considered for use in patients undergoing TAA.
